# “Real-world” analysis of battery longevity of implantable cardioverter-defibrillators: an in-depth analysis of a prospective defibrillator database

**DOI:** 10.1186/s12872-023-03653-6

**Published:** 2023-12-12

**Authors:** Diogo de Almeida Fernandes, Natália António, Pedro A. Sousa, Leonor Preto, Marta Madeira, Luís Elvas, Lino Gonçalves

**Affiliations:** 1 Department of Cardiology, Serviço de Cardiologia, Coimbra Hospital and University Centre (CHUC), Centro Hospitalar e Universitário de Coimbra, Praceta Professor Mota Pinto, 3004-561 Coimbra, Portugal; 2 Faculty of Medicine, University of Coimbra, Azinhaga de Santa Comba, 3000-548 Coimbra, Portugal; 3 Faculty of Medicine, ICBR, University of Coimbra, Azinhaga de Santa Comba, 3000-548 Coimbra, Portugal

**Keywords:** Implantable cardioverter-defibrillator, Longevity, Battery, Implantable devices

## Abstract

**Background:**

There is a lack of evidence regarding contemporary implantable cardioverter-defibrillator (ICD) battery longevity. Our aim was to assess battery longevity in ICDs in a real-world setting.

**Methods:**

Retrospective cross-sectional single center study of a prospectively collected database of consecutive patients who underwent ICD implantation from January 2010 to December 2015. Clinical data and battery longevity of all manufacturers were collected.

**Results:**

A total of 351 patients (84.6% males, mean age of 61 ± 12 years) were included in the study (292 VVI; 6 VDD; 53 DDD). All manufacturers (Abbott, Biotronik, Boston, Medtronic and Microport) were equally represented in the study (p = 0.110). Median battery longevity was 10.8 years (11 years for VVI and 8.5 for DDD). After a follow-up time of 5 years, 98% of VVI and DDD were still in service (vs. industry-projected longevity of 98%). During this time, 89 patients (25.4%) underwent device replacement − 69 patients (77.5%) due to battery depletion, 6 patients due to infection, 3 patients due to dysfunction and 13 patients due to upgrade to CRT-D. Patients with Medtronic or Biotronik ICDs had a greater probability of being replaced earlier due to battery depletion (Biotronik HR 6.87, 95% CI 2.54–18.58, p < 0.001; Medtronic HR 6.08, 95% CI 2.45–15.06 p < 0.001).

**Conclusions:**

VVI and DDD ICD battery longevity matched industry-projected longevity after 5 years of follow-up. Medtronic and Biotronik ICDs appeared to have an earlier battery depletion. Further randomized studies are required to ensure optimal care.

**Supplementary Information:**

The online version contains supplementary material available at 10.1186/s12872-023-03653-6.

## Introduction

The implantable cardioverter-defibrillator (ICD) has become an indispensable tool in the primary and secondary prevention of sudden cardiac death (SCD). The number of indications for an ICD has vastly increased and its impact on cardiac morbidity and mortality is now universally recognized [1, 2].

Despite all its proven benefits, this technological advance entails very high costs [3]. The implantation of an ICD is often considered a lifelong therapy and since patient survival often exceeds device lifetime, device replacement is frequent. Therefore, two major aspects arise regarding the longevity of these products: the impact on patients’ lives and the effective cost to public health systems [4]. Some studies have analyzed which factors can influence the lifespan of devices in clinical practice, including the manufacturer and type of device. Nevertheless, their results are inconsistent [1, 4–11]. Moreover, most manufacturers project the longevity of their ICDs at 5–9 years [12–16]. However, “real-world” battery longevity data is scarce. Few studies have included devices released after 2010 and the follow-up period of this subset of patients was short [1].

Our goal was to assess contemporary battery longevity of ICDs in a “real-world” setting.

## Materials & Methods

### Study design and Setting

Retrospective, single center study of a prospectively constructed database of consecutive patients referred for ICD implantation from 2010 to 2015 in a tertiary referral center. Procedural endpoints and long-term follow-up were assessed.

This study was approved by the local Ethics Committee (Approval number 121/CES; OBS.SF.023-2022). The ethical principles from the Declaration of Helsinki were followed and respected. Due to the retrospective nature of the study, informed consent was waived.

### Patient eligibility criteria

Patients were eligible for inclusion in the study if they had an indication for ICD implantation, irrespective of the pacing mode, according to current guidelines [2].

To achieve greater homogeneity, patients with previous devices or an indication for cardiac resynchronization therapy were excluded. Patients aged < 18 years or with subcutaneous ICD were also excluded from the analysis.

### Data collection

Baseline data, clinical and laboratory variables and ICD characteristics (such as type of ICD, manufacturer and device model) were collected from a prospective database. The choice of manufacturer and model was up to the attending cardiologist as all manufacturers were available from the beginning of the studied period.

Follow-up data were retrieved from the internal database, outpatient clinical and emergency department admissions records as well as device monitoring consultation. All ICD replacements were identified and their replacement date and reason (elective replacement indicator [ERI], device dysfunction, early extraction and upgrade to another type of device) were assessed. The average percentage of pacing (calculated as the average of atrial and ventricular pacing divided by 2 for DDDs and the simple average of ventricular pacing for VVIs) was ascertained. The total number of shocks delivered (appropriate and inappropriate) and antitachycardia pacing (ATP) therapies were also surveyed. Sensing and pacing thresholds as well as impedance of leads from depleted generators were recorded. Device or pocket infection, heart transplant and mortality during follow-up were also evaluated. All remaining devices were censored at the date of the last database access. The date of last access of clinical follow-up was March 05, 2022.

### Study endpoints

The primary endpoint was ICD battery longevity, defined as time from implantation to replacement due to ERI.

The secondary endpoint was longevity according to manufacturer.

### Industry-projected longevity

Data was collected from product performance reports (PPRs) for all generators included [12–16]. Survival probability was retrieved at yearly increments from the reports for all generators in our sample and the average of these probabilities was calculated. This result was used as the overall industry-projected longevity and compared to the survival probability of our sample.

### Statistical analysis

Analysis was performed with the use of IBM® SPSS® 26 and MedCalc® statistical software 19.6.3. Categorical variables are presented as frequencies and percentages. Continuous variables are expressed as means ± standard deviation or medians and interquartile ranges (IQR) for variables with or without normal distribution, respectively. Normal distribution was verified through the Kolmogorov-Smirnov test or by Skewness and Kurtosis measurements (maximum tolerated interval of -1 to 1). Bivariate analysis was performed using the χ2 test for categorical variables and t-test/ANOVA or Kruskal-Wallis test for continuous variables as appropriate.

Device longevity was defined as time in years from implantation to replacement due to ERI. Kaplan-Meier survival analysis was used to calculate median longevity by ICD type and manufacturer. Furthermore, comparison with industry-projected longevity was performed. Hazard ratios (HR), confidence intervals (95% CI), and p-values were calculated using the Cox proportional-hazards model, with adjustment for relevant variables (manufacturer, type of ICD, number of shocks, ATPs and pacing percentage) [1, 4, 5, 17, 18]. ICD replacements due to ERI battery status were considered events. Patients who were transplanted, died, underwent device upgrade or removal due to infection were censored at that respective date. ICDs still in service were censored at the date of last database access. Longevity was determined using Kaplan-Meier curves. Statistical significance was accepted for p values < 0.05.

### Patient and public involvement

Patients were not involved in the design or conduction of this study.

## Results

### Patient and ICD characteristics

**Fig. 1:**
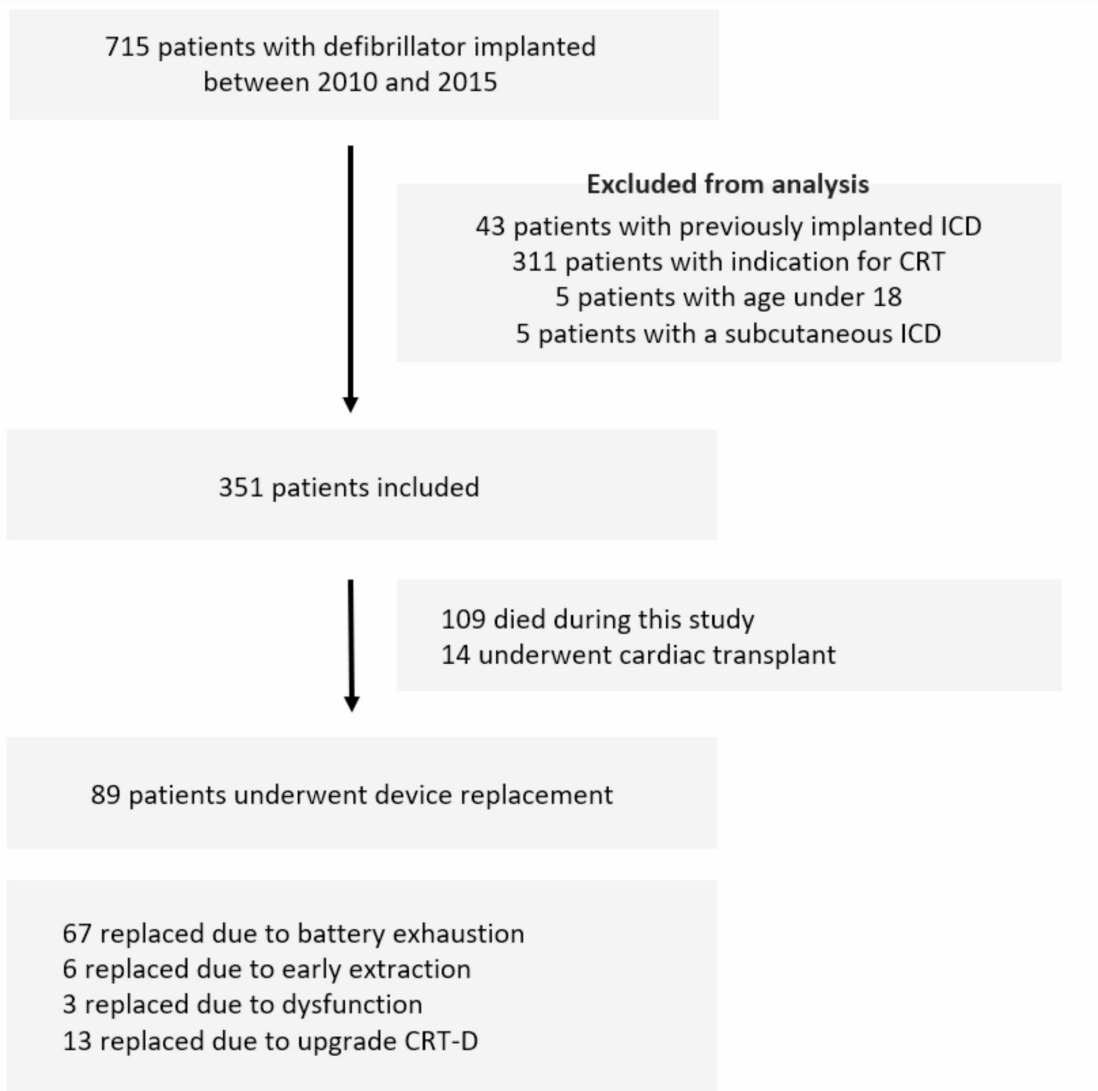
Flowchart of patient selection

The final study sample included 351 patients (84.6% men, mean age of 60.6 ± 11.9 years) (Fig. 1). It was found that 109 patients (31.1%) died and 13 patients (4.0%) underwent heart transplantation before ICD battery end of life. ICDs were implanted in the majority of the patients for primary prevention (79.8%). Single chamber devices were the most commonly implanted type (83.2%). Baseline characteristics are depicted in Table 1.

**Table 1:** Baseline characteristics

	Total		Not replaced		Replaced		P-value
Sample - n (%)	351		284	(80.9%)	67	(19.1%)	-
Male - n (%)	297	(84.6%)	238	(83.8%)	59	(89.1%)	0.385^§^
Age - years	60.6 ± 11.9		61.4 ± 11.8		56.9 ± 11.8		0.542^†^
Body mass index - Kg/cm^2^	27.3 ± 4.5		27.2 ± 4.5		28.1 ± 4.7		0.481^†^
Heart failure - n (%)	255	(72.6%)	213	(75.0%)	42	(62.7%)	**0.042** ^§^
LVEF - %	31.4 ± 11.0		31.1 ± 11.1		32.6 ± 10.6		0.445^†^
Atrial fibrillation - n (%)	124	(35.3%)	99	(34.9%)	25	(37.3%)	0.552^§^
Diabetes mellitus - n (%)	130	(37.0%)	104	(36.6%)	26	(38.8%)	0.410^§^
Hypertension - n (%)	226	(64.4%)	182	(65.1%)	41	(61.2%)	0.535^§^
Dyslipidemia - n (%)	258	(73.5%)	211	(74.3%)	47	(70.1%)	0.980^§^
Glomerular filtration rate – ml/min	78.2 ± 26.4		76.7 ± 26.5		84.7 ± 25.5		**0.043** ^†^
Primary prevention - n (%)	280	(79.8%)	226	(79.6%)	54	(80.6%)	0.983^§^
Indication - n (%) DCM HCM ICM Other	79 38 190 44	(22.5%) (10.8%) (54.1%) (12.5%)	69 24 157 34	(24.3%) (8.4%) (55.3%) (12.0%)	10 14 13 10	(14.9%) (20.9%) (49.3%) (14.9%)	**0.010** ^§^ 0.084^§^ **0.004** ^§^ 0.286^§^ 0.255^§^
ICD manufacturer - n (%) Abbott/St Jude Biotronik Boston/Guidant Medtronic Microport/Sorin	67 68 78 84 54	(19.1%) (19.4%) (22.2%) (23.9%) (15.4%)	60 49 70 56 49	(21.1%) (17.3%) (24.6%) (19.7%) (17.3%)	7 19 8 28 5	(10.4%) (28.4%) (11.9%) (41.8%) (7.5%)	**< 0.001** ^§^ **0.045** ^§^ **0.039** ^§^ **0.024** ^§^ **< 0.001** ^§^ **0.046** ^§^
Type of ICD - n (%) VVI VDD DDD	292 6 53	(83.2%) (1.7%) (15.1%)	243 4 37	(85.6%) (1.4%) (13.0%)	49 2 16	(73.1%) (3.0%) (23.9%)	**0.049** ^§^ **0.014** ^§^ 0.332* **0.026** ^§^
Shocks - n (%)	69	(19.7%)	58	(20.4%)	11	(16.4%)	0.423^§^
Number of shocks	0 (0–46)		0 (0–46)		0 (0–12)		0.291^#^
ATP - n (%)	56	(16.0%)	45	(15.8%)	11	(16.4%)	0.982^§^
Number of ATP	0 (0–56)		0 (0–45)		0 (0–56)		0.785^#^
Pacing - n (%) 0 1–25 26–50 51–75 76–99 100	271 41 13 4 13 9	(77.2%) (14.3%) (4.5%) (1.4%) (4.5%) (3.1%)	228 33 7 2 9 5	(80.3%) (11.6%) (2.5%) (0.7%) (3.2%) (1.8%)	43 8 6 2 4 4	(64.2%) (11.9%) (9.0%) (3.0%) (6.0%) (6.0%)	**0.047** ^§^ **0.033** ^§^ 0.716^§^ 0.040* 0.206* 0.489* 0.106*

ATP – antitachycardia pacing; DCM – Non-ischemic dilated cardiomyopathy; HCM – Hypertrophic cardiomyopathy; ICD – implantable cardioverter defibrillator; ICM – Ischemic cardiomyopathy; LVEF – Left ventricular ejection fraction

^§^Chi-squared; *Fisher; ^†^T-student; ^#^Mann-Whitney U

All manufacturers are equally represented in the study (p = 0.110) and no differences were found regarding pacing modes (p = 0.176) (Table 2). Additionally, all manufacturers were available from the first year of enrolment, with no significant differences between years (p = 0.572). The remaining characteristics of patients by manufacturer and ICD type are presented in Supplemental Tables 1 and 2 respectively. Defibrillator threshold testing was not performed on any patient.

**Table 2:** Manufacturers and pacing modes

Manufacturer	VVI	VDD	DDD	Total
Abbott/St Jude	63	0	4	67
Biotronik	55	6	7	68
Boston/Guidant	63	0	15	78
Medtronic	66	0	18	84
Microport/Sorin	45	0	9	54
Total	**292**	**6**	**53**	**351**

### Primary endpoint

Overall median follow-up time was 7.5 years (IQR 4.38). During this period, 89 patients (25.4%) underwent device replacement. Of these, 67 were due to battery depletion. (Fig. 1).

Patients with battery depletion were less likely to have heart failure (p = 0.042), more likely to have a diagnosis of hypertrophic cardiomyopathy (p = 0.004), had higher glomerular filtration rates (p = 0.043), higher implantation of Medtronic (p < 0.001) and Biotronik (p 0.039) ICDs as well as more dual chamber pacing mode (p = 0.026) compared to patients without battery depletion.

Overall median longevity was 10.8 years. Median longevity by manufacturer and type of ICD determined by survival curves is shown in Table 3.

**Table 3:** Median battery longevity by manufacturer and ICD type

Manufacturer	VVI	DDD	Overall
Abbott/St Jude	11.0	8.1	11.0
Biotronik	9.1	7.7	9.1
Boston/Guidant	11.0	11	11.0
Medtronic	9.4	7.7	9.3
Microport/Sorin	10.0	7.5	10.0
Overall	**11.0**	**8.5**	**10.8**

**Fig. 2:**
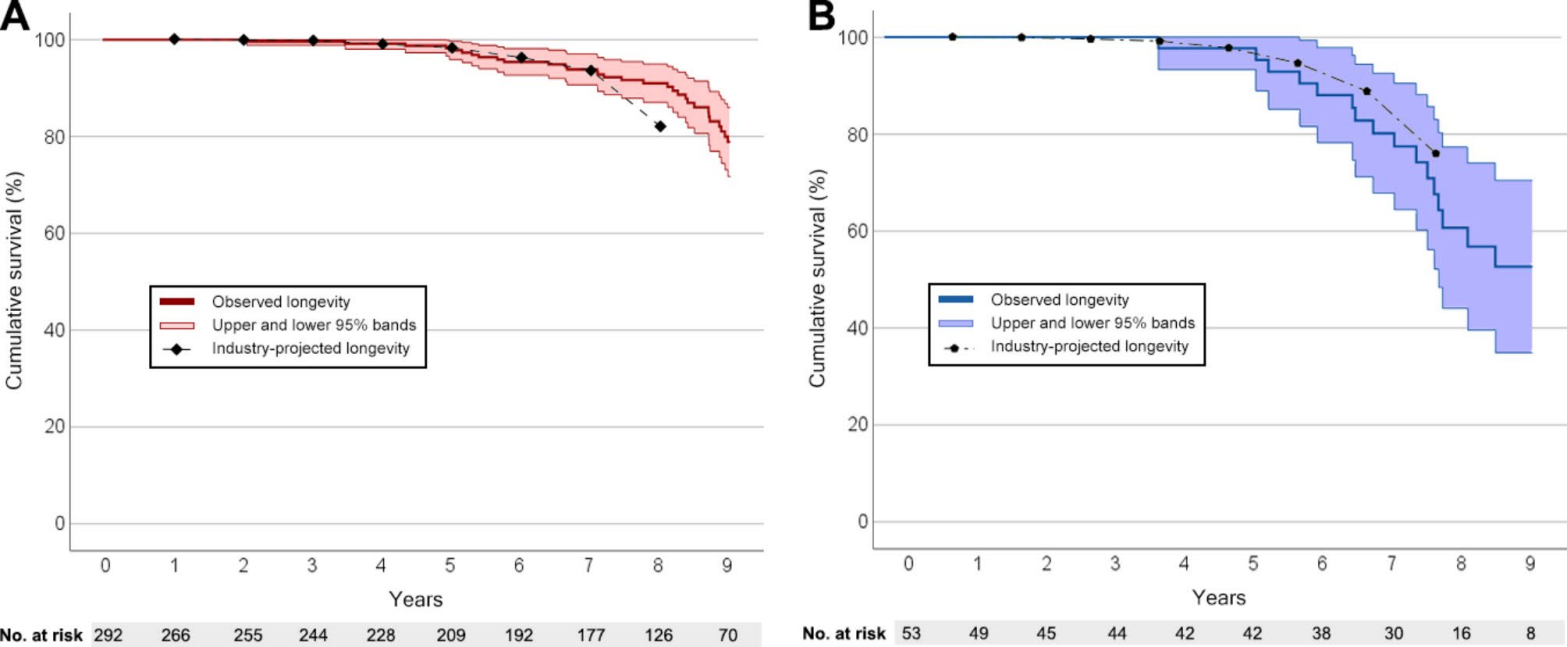
Kaplan-Meier curves of observed and industry-projected longevities. **A** – VVI. **B** – DDD

Regarding industry-projected longevity, our cohort showed a similar longevity to that reported in the product performance reports (PPRs) (Fig. 2). After a follow-up time of 5 years, 98% of VVI and DDD were still in service (vs. industry-projected longevity of 98%). These results persisted after 7 years of follow-up with 94% of VVI still in service (vs. industry-projected longevity of 93%) (Fig. 2A). Regarding DDD, our cohort showed a 81% survival of the devices in contrast to an industry-projected longevity of 89%. (Fig. 2B) Nevertheless, all estimates provided by the industry fell inside the 95% confidence interval.

**Fig. 3:**
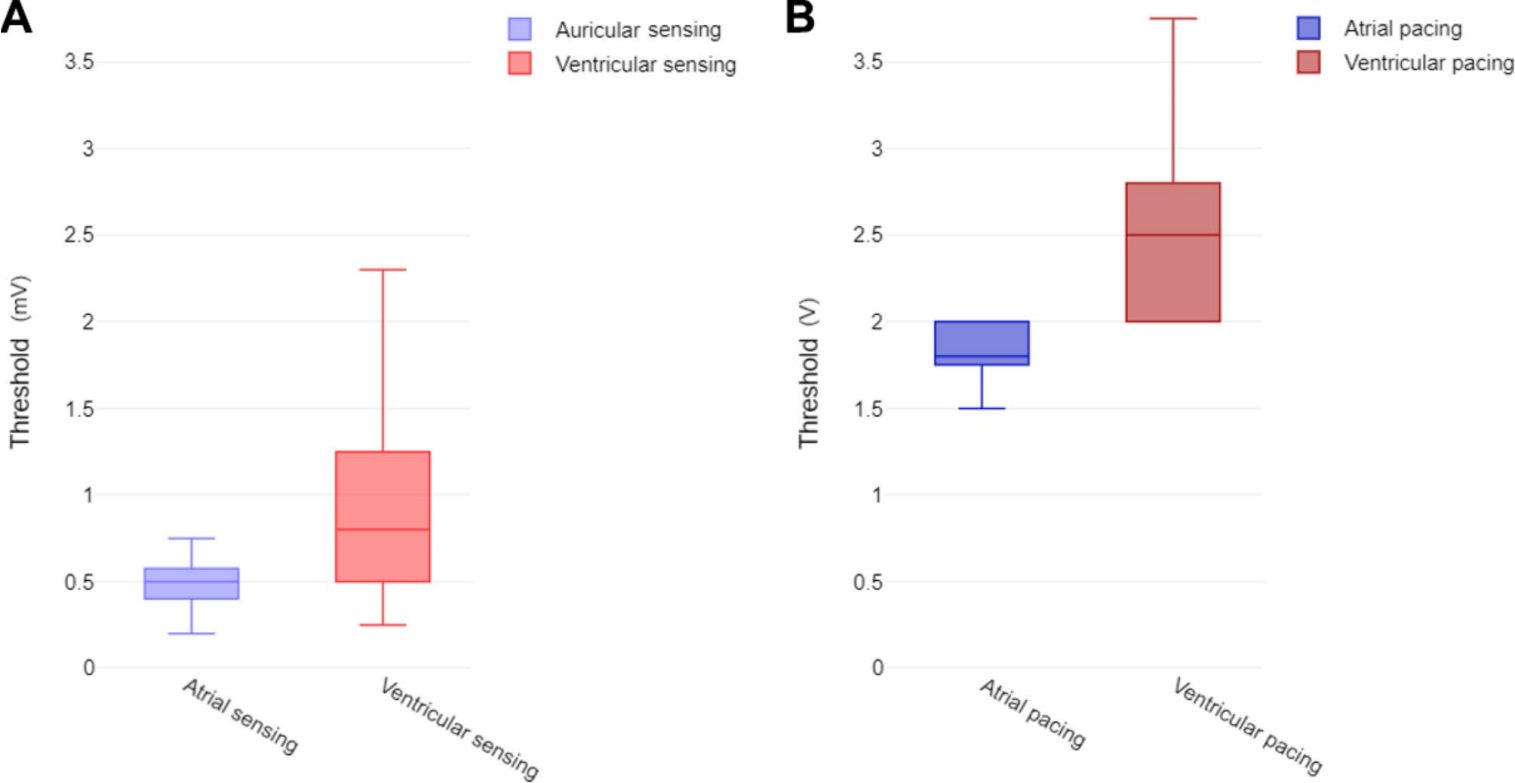
Parameters of depleted ICDs. **A** – Sensing thresholds. **B** – Pacing thresholds

Median atrial impedance was 551 Ohm (IQR 404) whereas ventricular impedance was 494 Ohm (IQR 446). Median atrial sensing and pacing thresholds for leads from depleted generators are shown in Fig. 3A and B.

### Secondary endpoint

**Fig. 4:**
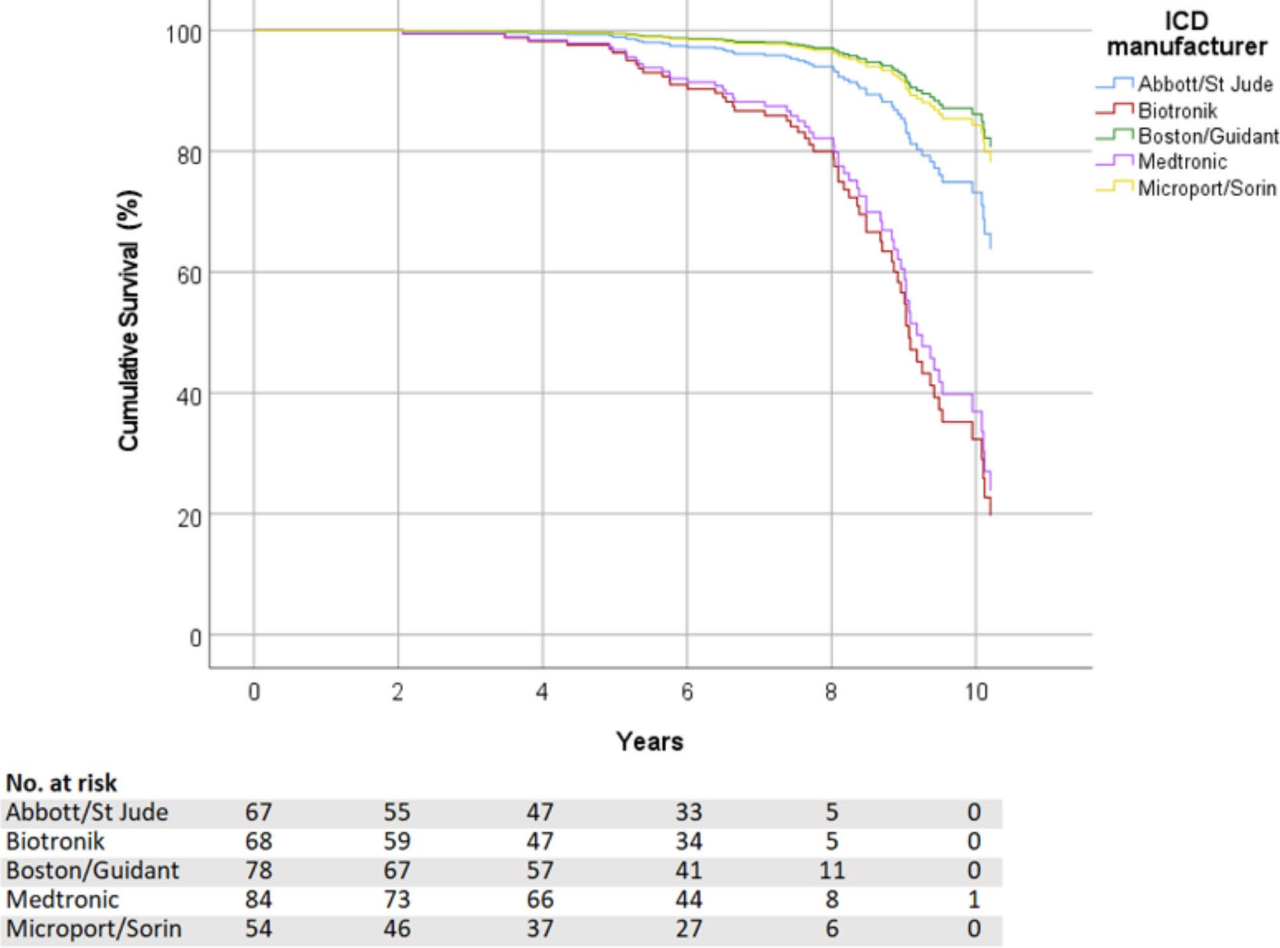
Kaplan-Meier survival curves for battery depletion in the different manufacturers

Battery longevity between manufacturers was statistically different (p < 0.001; Fig. 4). Compared to the other manufacturers, patients with Medtronic or Biotronik ICDs had a higher risk of being replaced earlier due to battery depletion even after adjustment for ATP therapies, shocks and other confounders (Table 4). ATP and shocks had a non-significant impact on longevity (p = 0.980 and p = 0.307 respectively) whereas pacing percentage was the only other significant influence besides manufacturer (p 0.047; HR 1.27; 95% CI 1.01–1.61).

**Table 4:** – Hazard ratio and confidence interval for battery depletion by manufacturer

Manufacturer	Abbott/St Jude	Biotronik	Boston/Guidant	Medtronic	Microport/Sorin
Abbott/St Jude	-	**p = 0.010** ***0.28 (0.11–0.74)***	p = 0.271 1.93 (0.60–6.26)	**p = 0.014** ***0.32 (0.13–0.79)***	p = 0.425 1.68 (0.47–6.05)
Biotronik	**p = 0.010** ***3.55 (1.36–9.31)***	-	**p < 0.001** ***6.87 (2.54–18.58)***	p = 0.719 1.13 (0.58–2.20)	**p = 0.002** ***5.99 (1.94–18.43)***
Boston/Guidant	p = 0.271 0.52 (0.16–1.67)	**p < 0.001** ***0.15 (0.05–0.39)***	-	**p < 0.001** ***0.17 (0.07–0.41)***	p = 0.831 0.87 (0.25–3.09)
Medtronic	**p = 0.014** ***3.14 (1.26–7.82)***	p = 0.719 0.89 (0.45–1.72)	**p < 0.001** ***6.08 (2.45–15.06)***	-	**p = 0.002** ***5.29 (1.83–15.28)***
Microport/Sorin	p = 0.425 0.59 (0.17–2.13)	**p = 0.002** ***0.17 (0.05–0.52)***	p = 0.831 1.15 (0.32–4.08)	**p = 0.002** ***0.19 (0.07–0.55)***	-

*Note*: all hazard ratios presented are adjusted for confounders. Reference manufacturer is presented in the columns

## Discussion

To the best of our knowledge this is the first study that evaluates long-term follow-up of devices implanted solely in the last decade using real-world data and where all manufacturers were included. Our findings suggest that (1) single chamber and dual chamber ICD battery longevity is similar to the estimation provided by manufacturers and (2) Biotronik and Medtronic ICDs may present a higher risk of being replaced due to earlier battery depletion.

There are few articles published comparing the battery longevity of ICD devices from different manufacturers. Many of the models analyzed in previous studies have already been discontinued [7]. This makes scientific information about the current longevity of devices from different manufacturers even more scarce [8]. Previous studies reported an average battery longevity for VVIs of 5–7 years and DDDs of 5–6 years [1, 6, 7, 19]. At the present time, manufacturers estimate a longevity of 8 to 10 years in single-chamber ICDs and 6 to 9 years in double-chamber ICDs according to the product performance reports (PPRs) [12–16]. Industry-projected longevity at 5 and 7 years was similar to the longevity of the studied cohort. Overall battery longevity was 10.8 years. When analyzing by subtype, VVI longevity was slightly higher than previously reported, with a median battery longevity of 11.0 years. DDD median longevity was 8.5 years. This sharp contrast with previous studies is probably the result of improved battery and lead technologies [10].

In our population, patients with ICDs from Medtronic and Biotronik had a lower longevity, in line with previous studies [4, 6, 18]. These differences may be the result of lower battery capacities present in Medtronic and Biotronik devices [10]. A recent single study using PPRs concluded that Boston DDD-ICDs had longer battery longevity, whereas in VVI devices, Abbot and Medtronic’s were superior [9]. Nevertheless, longevity in PPRs may not represent real-world experiences as it is often overestimated due to underreporting as well as not all manufacturers available in Europe having been studied [6, 9, 11, 17]. Furthermore, there is great heterogeneity in the assumptions made by the manufacturers in order to estimate device longevity [10]. It is also worth noting that other studies have shown superior battery longevity of Medtronic devices [1, 7, 20]. This discrepancy between manufacturers does not seem to be justified by battery chemistry alone since Boston and Biotronik used preferably LiMnO2 batteries in contrast to the remaining manufacturers that use Li/SVO-CFx batteries. Our study takes into consideration the pacing percentage and ICD therapies, providing new information regarding devices implanted in the last decade that were previously unavailable. We hypothesize that these differences among studies may result from an asymmetric number of implantations among different manufacturers, differences in current battery and device technologies and lack of adjustment for device therapies.

It is clear that a shorter battery life contributes to morbidity and mortality [10]. Reinterventions have been linked to device-related infections and other complications [21]. As such, it is of the utmost importance that battery longevity be improved to minimize reinterventions. Ultimately, a lower rate of reinterventions may even culminate in a better quality of life and improved mental health. This is an essential issue in this population since anxiety and depression are particularly prevalent [2]. In fact, a National Institute of Health and Care Excellence (NICE) guidance document reports that battery longevity improvement is the main factor for more significant clinical benefit [10, 22]. An additional impact of reduced longevity is financial burden. As health-services around the world are increasingly under strain, greater longevity and consequently fewer reinterventions and complications are crucial in order to ensure their sustainability. It is paramount to have information about the newer devices because only then will it be possible to make an ICD choice with the best cost-benefit ratio [10]. Our study provides an update on the literature data and new information regarding newer devices released after 2010.

### Limitations

We acknowledge some limitations in the present study. First, this was a single-center retrospective study. However, this was mitigated since data was collected from our prospective database. Second, ICD indications differed significantly between manufacturers which may have led to asymmetries in therapies and pacing percentage. Thirdly, the number of patients included for each manufacturer in this study was insufficient to allow more robust conclusions. Nonetheless, the study size was comparable to other similar studies. Randomized, large-scale studies are required to confirm the results obtained in our study and to assess their clinical implications even though they are unlikely to be made. Fourthly, the sample is comprised mainly of men. This underrepresentation of women is probably justified by the high prevalence of ischemic heart disease in men. Two meta-analyses showed that women received less appropriate ICD therapies [23, 24]. Ultimately this fact could mean greater battery longevity even though it remains to be studied. Finally, we recognize that different ICD models of the same brand were analyzed, which have different intrinsic characteristics that may affect the longevity of the device [12–16]. Nevertheless, battery and lead technology as well as programming are similar throughout all devices of a given manufacturer and as such we believe the validity of these findings still holds.

## Conclusion

In conclusion, the battery longevity of single and dual chamber ICDs in the real-world appears to be in agreement with estimations provided by manufacturers. Further studies are required, given that these results have important clinical implications for both patients and hospitals.

### Electronic supplementary material

Below is the link to the electronic supplementary material.

**Supplemental table 1:** Baseline characteristics by manufacturer

**Supplemental table 2:** Baseline characteristics by ICD type

## Data Availability

All data generated or analysed during this study are included in this published article and its supplementary information files.

## List of abbreviations

ATP: antitachycardia pacing
CI: confidence interval
ERI: elective replacement indicator
HR: hazard ratio
ICD: Implantable cardioverter-defibrillator
IQR: interquartile ratio
NICE: National Institute of Health and Care Excellence
PPR: product performance reports
SCD: sudden cardiac death
