# Supplementary material for: “Real-world” analysis of battery longevity of implantable cardioverter-defibrillators: an in-depth analysis of a prospective defibrillator database
Source: BMC Cardiovasc Disord. 2023 Dec 12;23:609. doi: 10.1186/s12872-023-03653-6 (PMC10717854; doi:10.1186/s12872-023-03653-6)
Supplement: Supplementary file 1 — Supplemental table 1: Baseline characteristics by manufacturer [file 12872_2023_3653_MOESM1_ESM.docx]

Supplemental table 1 - Baseline characteristics by manufacturer

|  | Total | | Abbott/St Jude | | Biotronik | | Boston/Guidant | | Medtronic | | Microport/Sorin | | p-value |
| --- | --- | --- | --- | --- | --- | --- | --- | --- | --- | --- | --- | --- | --- |
| Sample - n (%) | 351 |  | 67 | (19.1%) | 68 | (19.4%) | 78 | (22.2%) | 84 | (23.9%) | 54 | (15.4%) | 0.110^§^ |
| Gender (male) - n (%) | 297 | (84.6%) | 56 | (83.6%) | 60 | (88.2%) | 65 | (83.3%) | 71 | (84.5%) | 45 | (83.3%) | 0.924^§^ |
| Age - years | 60.6 ± 11.9 | | 60.8 ± 12.0 | | 61.5 ± 9.39 | | 58.4 ± 11.68 | | 57.7 ± 14.1 | | 66.6 ± 9.1 | | **<0.001^*^** |
| Body mass index - Kg/cm^2^ | 26.6 (17.4-45.3) | | 24.4 (14.4-35.9) | | 26.1 (19.8-34.84) | | 27.1 (21.3-45.3) | | 28.3 (21.6-37.2) | | 27.2 (20.8-37.55) | | 0.276^#^ |
| Heart failure - n (%) | 255 | (72.6%) | 54 | (80.6%) | 55 | (80.9%) | 58 | (74.4%) | 51 | (60.7%) | 37 | (68.5%) | **0.026**^§^ |
| LVEF - % | 29.0 (10.0-70.0) | | 31.0 (10.0-70.0) | | 27 (15.0-61.0) | | 30.0 (14.0-65.0) | | 30.0 (13.0-65.0) | | 29.0 (15.0-53.0) | | 0.050^#^ |
| Atrial fibrillation - n (%) | 124 | (35.3%) | 32 | (55.2%) | 21 | (35.6%) | 22 | (33.8%) | 26 | (38.2%) | 23 | (47.9%) | 0.092^§^ |
| Diabetes mellitus - n (%) | 130 | (37.0%) | 18 | (32.1%) | 34 | (59.6%) | 21 | (34.4%) | 29 | (44.6%) | 28 | (62.2%) | **0.002^§^** |
| Hypertension - n (%) | 226 | (64.4%) | 46 | (82.1%) | 46 | (79.3%) | 43 | (69.4%) | 51 | (78.5%) | 40 | (88.9%) | 0.166^§^ |
| Dyslipidemia - n (%) | 258 | (73.5%) | 52 | (86.7%) | 59 | (92.2%) | 50 | (76.9%) | 52 | (74.3%) | 45 | (93.8%) | **0.006^§^** |
| Glomerular filtration rate – ml/min | 78.2 ± 26.4 | | 79.5 ± 27.9 | | 74.29 ± 27.8 | | 80.1 ± 26.5 | | 80.5 ± 25.90 | | 75.7 ± 23.8 | | 0.648^*^ |
| Primary prevention - n (%) | 280 | (79.8%) | 15 | (22.7%) | 11 | (16.4%) | 11 | (14.3%) | 18 | (21.7%) | 12 | (22.2%) | 0.610^§^ |
| Indication - n (%)  DCM  HCM  ICM  Other | 79  38  190  44 | (22.5%)  (10.8%)  (54.1%)  (12.5%) | 14  4  40  8 | (21.2%)  (6.1%)  (60.6%)  (12.1%) | 10  3  47  5 | (15.4%)  (4.6%)  (72.3%)  (7.7%) | 19  11  40  7 | (24.7%)  (14.3%)  (51.9%)  (9.1%) | 25  16  29  13 | (30.1%)  (19.3%)  (34.9%)  (15.7%) | 11  4  34  5 | (20.4%)  (7.4%)  (63.0%)  (9.3%) | **0.006**^§^ |
| ICD type – n (%)  VVI  VDD  DDD | 292  6  53 | (83.2%)  (1.7%)  (15.1%) | 63  0  4 | (94.0%)  (0.0%)  (6.0%) | 55  6  7 | (80.9%)  (8.8%)  (10.3%) | 63  0  15 | (80.8%)  (0.0%)  (19.2%) | 66  0  18 | (78.6%)  (0.0%)  (21.4%) | 45  0  9 | (83.3%)  (0.0%)  (16.7%) | 0.176^§^ |
| Shocks - n (%) | 69 | (19.7%) | 18 | (27.7%) | 13 | (22.0%) | 15 | (20.5%) | 13 | (16.7%) | 10 | (19.6%) | 0.605^§^ |
| Number of shocks | 0 (0-46) | | 0 (0-15) | | 0 (0-10) | | 0 (0-46) | | 0 (0-35) | | 0 (0-4) | | 0.489^#^ |
| ATP - n (%) | 56 | (16.0%) | 13 | (20.3%) | 12 | (20.3%) | 15 | (20.8%) | 12 | (16.0%) | 4 | (8.2%) | 0.367^§^ |
| Number of ATP | 0 (0-56) | | 0 (0-8) | | 0 (0-56) | | 0 (0-24) | | 0 (0-20) | | 0 (0-45) | | 0.439^#^ |
| Pacing - n (%)  0  1-25  26-50  51-75  76-99  100 | 271  41  13  4  13  9 | (77.2%)  (14.3%)  (4.5%)  (1.4%)  (4.5%)  (3.1%) | 44  8  3  0  1  0 | (78.6%)  (14.3%)  (5.4%)  (0.0%)  (1.8%)  (0.0%) | 38  8  2  1  3  1 | (71.7%)  (15.1%)  (3.8%)  (1.9%)  (5.7%)  (1.9%) | 47  8  2  0  4  5 | (71.2%)  (12.1%)  (3.0%)  (0.0%)  (5.7%)  (7.6%) | 47  11  2  2  3  2 | (70.1%)  (16.4%)  (3.0%)  (3.0%)  (4.5%)  (3.0%) | 30  6  4  1  2  1 | (68.2%)  (13.6%)  (9.1%)  (2.3%)  (4.5%)  (2.3%) | 0.794^§^ |

^§^Chi-squared; ^*^ANOVA; ^#^Kruskal-Wallis

ATP – antitachycardia pacing; DCM – Non-ischemic dilated cardiomyopathy; HCM – Hypertrophic cardiomyopathy; ICD – implantable cardioverter defibrillator; ICM – Ischemic cardiomyopathy; LVEF – Left ventricular ejection fraction
