# Supplementary material for: “Real-world” analysis of battery longevity of implantable cardioverter-defibrillators: an in-depth analysis of a prospective defibrillator database
Source: BMC Cardiovasc Disord. 2023 Dec 12;23:609. doi: 10.1186/s12872-023-03653-6 (PMC10717854; doi:10.1186/s12872-023-03653-6)
Supplement: Supplementary file 2 — Supplemental table 2: Baseline characteristics by ICD type [file 12872_2023_3653_MOESM2_ESM.docx]

Supplemental table 2 - Baseline characteristics by ICD type

|  | Total | | VVI | | DDD | | p value |
| --- | --- | --- | --- | --- | --- | --- | --- |
| Sample - n (%) | 351 |  | 292 | (83.2%) | 53 | (15.1%) |  |
| Gender (male) - n (%) | 297 | (84.6%) | 251 | (86.0%) | 41 | (77.4%) | 0.110^§^ |
| Age - years | 60.6 ± 11.9 | | 60.6 ± 11.7 | | 59.9 ± 13.5 | | 0.667^*^ |
| Body mass index - Kg/cm^2^ | 26.6 (17.4-45.3) | | 26.6 (17.4-45.3) | | 26.1 (19.8-34.6) | | 0.858^#^ |
| Heart failure - n (%) | 255 | (72.6%) | 228 | (78.1%) | 22 | (41.5%) | **<0.001^§^** |
| LVEF - % | 29.0 (10.0-70.0) | | 29.0 (10.0-70.0) | | 35.0 (20.0-65.0) | | **<0.001^#^** |
| Atrial fibrillation - n (%) | 124 | (35.3%) | 104 | (41.4%) | 16 | (38.1%) | 0.684^§^ |
| Diabetes mellitus - n (%) | 130 | (37.0%) | 116 | (48.5%) | 11 | (27.5%) | **0.013^§^** |
| Hypertension - n (%) | 226 | (64.4%) | 194 | (80.5%) | 28 | (70.0%) | 0.131^§^ |
| Dyslipidemia - n (%) | 258 | (73.5%) | 224 | (86.5%) | 28 | (66.7%) | **0.001^§^** |
| Glomerular filtration rate – ml/min | 78.2 ± 26.4 | | 77.8 ± 26.59 | | 81.5 ± 26.1 | | 0.409^*^ |
| Primary prevention - n (%) | 280 | (79.8%) | 53 | (18.4%) | 13 | (24.5%) | 0.300^§^ |
| Indication - n (%)  DCM  HCM  ICM  Other | 79  38  190  44 | (22.5%)  (10.8%)  (54.1%)  (12.5%) | 73  18  164  31 | (25.5%)  (6.3%)  (57.3%)  (10.8%) | 5  20  21  7 | (9.4%)  (37.7%)  (39.6%)  (13.2%) | **<0.001^§^** |
| ICD manufacturer - n (%)  Abbott/St Jude  Biotronik  Boston/Guidant  Medtronic  Microport/Sorin | 67  68  78  84  54 | (19.1%)  (19.4%)  (22.2%)  (23.9%)  (15.4%) | 63  55  63  66  45 | (21.6%)  (18.8%)  (21.6%)  (22.6%)  (15.4%) | 4  7  15  18  9 | (7.5%)  (13.2%)  (28.3%)  (34.0%)  (17.0%) | 0.605^§^ |
| Shocks - n (%) | 69 | (19.7%) | 61 | (22.4%) | 8 | (16.7%) | 0.371^§^ |
| Number of shocks | 0 (0-46) | | 0 (0-46) | | 0 (0-35) | | 0.481^#^ |
| ATP - n (%) | 56 | (16.0%) | 49 | (18.4%) | 6 | (13.0%) | 0.382^§^ |
| Number of ATP | 0 (0-56) | | 0 (0-56) | | 0 (0-24) | | 0.599^#^ |
| Pacing - n (%)  0  1-25  26-50  51-75  76-99  100 | 271  41  13  4  13  9 | (77.2%)  (14.3%)  (4.5%)  (1.4%)  (4.5%)  (3.1%) | 195  32  0  9  2  3 | (80.9%)  (13.3%)  (0.0%)  (3.7%)  (0.8%)  (1.2%) | 8  7  9  4  2  10 | (20.0%)  (17.5%)  (22.5%)  (10.0%)  (5.0%)  (25.0%) | **<0.001^§^** |

^§^Chi-squared; ^†^T-student; ^#^Mann-Whitney U

VDD were not included in this analysis due to small sample size (n=6).

ATP – antitachycardia pacing; DCM – Non-ischemic dilated cardiomyopathy; HCM – Hypertrophic cardiomyopathy; ICD – implantable cardioverter defibrillator; ICM – Ischemic cardiomyopathy; LVEF – Left ventricular ejection fraction
